# Ischemic biomarker heart-type fatty acid binding protein (hFABP) in acute heart failure - diagnostic and prognostic insights compared to NT-proBNP and troponin I

**DOI:** 10.1186/s12872-015-0026-0

**Published:** 2015-06-14

**Authors:** Ursula Hoffmann, Florian Espeter, Christel Weiß, Parviz Ahmad-Nejad, Siegfried Lang, Martina Brueckmann, Ibrahim Akin, Michael Neumaier, Martin Borggrefe, Michael Behnes

**Affiliations:** 1 First Department of Medicine, University Medical Centre Mannheim (UMM), Faculty of Medicine Mannheim, University of Heidelberg, Theodor-Kutzer-Ufer 1-3, 68167 Mannheim, Germany; 2 Department of Statistical Analysis, Faculty of Medicine Mannheim, University of Heidelberg, Mannheim, Germany; 3 Institute for Microbiology and Laboratory Medicine, HELIOS Klinikum Wuppertal, University of Witten/Herdecke, Wuppertal, Germany; 4 Boehringer Ingelheim GmbH & Co. KG, Ingelheim am Rhein, Germany; 5 Faculty of Medicine Mannheim, University of Heidelberg, Mannheim, Germany; 6 Institute for Clinical Chemistry, University Medical Centre Mannheim (UMM), Faculty of Medicine Mannheim, University of Heidelberg, Mannheim, Germany

**Keywords:** Acute heart failure, hFABP, Mortality, NT-proBNP, Prognosis, Rehospitalisation, Troponin I

## Abstract

**Background:**

To evaluate diagnostic and long-term prognostic values of hFABP compared to NT-proBNP and troponin I (TnI) in patients presenting to the emergency department (ED) suspected of acute heart failure (AHF).

**Methods:**

401 patients with acute dyspnea or peripheral edema, 122 suffering from AHF, were prospectively enrolled and followed up to 5 years. hFABP combined with NT-proBNP versus NT-proBNP alone was tested for AHF diagnosis. Prognostic value of hFABP versus TnI was evaluated in models predicting all-cause mortality (ACM) and AHF related rehospitalization (AHF-RH) at 1 and 5 years, including 11 conventional risk factors plus NT-proBNP.

**Results:**

Additional hFABP measurements improved diagnostic specificity and positive predictive value (PPV) of sole NT-proBNP testing at the cutoff <300 ng/l to “rule out” AHF. Highest hFABP levels (4th quartile) were associated with increased ACM (hazard ratios (HR): 2.1–2.5; *p* = 0.04) and AHF-RH risk at 5 years (HR 2.8–8.3, *p* = 0.001). ACM was better characterized in prognostic models including TnI, whereas AHF-RH was better characterized in prognostic models including hFABP. Cox analyses revealed a 2 % increase of ACM risk and 3–7 % increase of AHF-RH risk at 5 years by each unit increase of hFABP of 10 ng/ml.

**Conclusions:**

Combining hFABP plus NT-proBNP (<300 ng/l) only improves diagnostic specificity and PPV to rule out AHF. hFABP may improve prognosis for long-term AHF-RH, whereas TnI may improve prognosis for ACM.

**Trial registration:**

ClinicalTrials.gov identifier: NCT00143793.

## Background

Human heart-type fatty acid binding protein (hFABP) is a 15 kDa small protein consisting of 132 amino acids [1]. It belongs to the FABP superfamily being characterized by relative tissue specificity [2]. hFABP is located primarily in the heart constituting 5–15 % of the cytosolic protein pool [3] and is assigned to transport fatty acids towards the mitochondria for ß-oxidation and energy expenditure [3–5]. Moreover it protects against free radical accumulation during myocardial ischemia [6–8] and influences signal transduction pathways for gene expression via peroxisome proliferator-activated receptors (PPAR) [9]. In healthy humans the normal range of hFABP in serum or plasma has been reported to vary between 0.0 and 5.5 ng/ml [10, 11]. Fatty acids represent the main source of energy in the heart accounting for 10 % of the total body turnover of fatty acids [4, 12–14].

The diagnostic value of hFABP has comprehensively been evaluated in patients suffering from acute myocardial infarction (AMI). Here, varying diagnostic area under the receiver-operating characteristic (ROC) curves and diagnostic goodness criteria (such as sensitives, specifities, positive and negative predictive values (NPV/PPV)) have been reported for hFABP measurements [15, 16]. Accordingly, combining hFABP with cardiac troponins revealed conflicting results [17–22]. In contrast, low specifities and PPVs have been reported for NT-proBNP to reliably diagnose acute heart failure (AHF). Therefore new biomarkers are increasingly focussed to improve both the diagnostic and prognostic assessement of AHF patients [23, 24]. hFABP was shown to be associated with chronic heart failure patients (CHF), whereas minor reports indicate a diagnostic role of hFABP in children developing concomitant acute heart failure (AHF) during pneumonia [25–28]. However, hFABP levels have never been primarily evaluated in patients suspected of AHF.

The present study evaluates the diagnostic and long term prognostic value of additional hFABP measurements compared to NT-proBNP and TnI in patients presenting with symptoms of acute dyspnea or peripheral edema to the emergency department.

## Methods

### Study patients, design and data collection

The present study represents a post hoc analysis of a specimen repository from patients enrolled in the Mannheim NT-proBNP Study (MANPRO, clinicaltrials.gov identifier: NCT00143793) [29], which was conducted as a single-centre prospective controlled study at the University Medical Centre Mannheim (UMM), Germany. The study was carried out according to the principles of the declaration of Helsinki and was approved by the medical ethics commission II of the Medical Faculty Mannheim, University of Heidelberg, Germany. Informed consent was obtained from all participating patients or their legal representatives.

Briefly, patients with symptoms of acute dyspnea and/or peripheral edema presenting to the emergency department were consecutively included from August 2005 until March 2006 and the underlying diseases such as AHF were diagnosed. Patients suffering from severe renal disease (defined as serum creatinine level greater than 2.8 mg/dl), anemia (hemoglobin concentrations below 8.0 g/dl), obvious traumatic causes of dyspnea, pregnancy, with a status after immediate cardiopulmonary resuscitation, participation in another clinical trial and patients with age under 18 years were excluded [29, 30].

### Diagnosis of acute heart failure

The investigators of the study were neither involved in therapeutic decisions nor in decisions regarding clinical examinations. To determine the main diagnosis of each patient, an independent study physician had unrestricted access to the records of the patients, but was blinded to the results of the biomarker measurements. Based on this approach all patients were classified into two categories: 1) Symptomatic patients because of AHF, 2) symptomatic patients due to any cause except for AHF.

Diagnosis of AHF was based on European Guidelines for the diagnosis of AHF [31, 32]. AHF diagnosis being a decompensated CHF or de-novo AHF, was based on the acute development of typical symptoms. Additionally, specific clinical signs made AHF diagnosis even more favourable, such as pulmonary rales, elevated jugular venous pressure or hepatojugular reflux. At least one of the following technical findings substantiated the diagnosis of AHF, such as radiographic evidence of pulmonary congestion and edema, abnormalities on the ECG (i.e. supraventricular tachycardia, ventricular arrhythmias, myocardial ischemia or infarction) or evidence of left ventricular (LV) systolic dysfunction (defined as LVEF <55 %) or diastolic dysfunction as assessed by echocardiography (see below). Patients not fulfilling AHF criteria were collected in the “no AHF” group. Diagnoses were not based on biomarker levels (such as hFABP or NT-proBNP) because patients and physicians were blinded to biomarker results.

Severity of symptoms were classified according to the functional New York Heart Association (NYHA) classification and structural ABCD classification of the American College of Cardiology/American Heart Association [31, 33]. Standard two-dimensional and colour Doppler imaging was performed by independent cardiologists during routine clinical care [30]. Parameters of LV systolic function comprised LV ejection fraction (Simpson’s biplane) (LVEF) and fractional shortening by parasternal M-mode. A LVEF of <55 % was defined as LV systolic dysfunction. Diastolic function was routinely assessed by the E/A ratio during sinus rhythm (the ratio of the maximum velocities of early (E) to atrial (A) LV filling determined by pulsed-wave Doppler of the mitral valve), deceleration time, isovolumetric relaxation time and ratio of E/E’ (which is used to estimate an increase in LV diastolic pressure, measured at the basal septum). Definitions of diastolic dysfunction corresponded to E/E’ >15 or stages 1, 2 and 3 [34].

### Measurements of hFABP, NT-proBNP and troponin I

All samples were obtained by venipuncture into serum and ammonium heparin tubes for biomarker measurements, immediately at presentation to the emergency department. Within 30 min all blood samples were centrifuged at 2000 g for 10 min. Plasma was separated, aliquoted, frozen and stored at −80 °C.

hFABP was measured in serum, NT-proBNP and troponin I (TnI) were measured in ammonium heparin plasma in all 401 patients. hFABP measurement was performed with a commercially available immunoassay/ELISA (hFABP ELISA Catalog Number EA-0305, Signosis Inc., Sunnyvale, USA) [35]. hFABP was shown to be a stable protein even after repeated freezing and thawing [36]. NT-proBNP measurement was performed with a commercially available immunoassay on the Dimension® RxL clinical chemistry system (Flex reagent cartridge PBNP, Dimension System, Dade Behring Ltd., Atterbury Milton Keynes, United Kingdom) as previously described [29]. Contemporary sensitive TnI was measured with the SIEMENS Dimension® Vista intelligent lab system for contemporary sensitive cardiac troponin I testing. The lowest detection limit of the assay is 0.015 ng/ml. The 99th percentile measured at a healthy reference population is 0.045 ng/ml with a coefficient of variation (CV) of 10 % [37].

### Study endpoints

The first endpoint tested was whether the diagnostic value of combined hFABP plus NT-proBNP was comparable to NT-proBNP alone for diagnosis of AHF.

The second endpoint was to test the prognostic value of hFABP in patients admitted to the emergency department with symptoms of acute dyspnea and/or edema. Two prognostic outcomes were considered: all-cause mortality and AHF-related rehospitalization at 1 and 5 years.

Follow-up was performed in three successive steps: Firstly, our in-hospital electronic records were screened with regard to in-hospital all-cause mortality, AHF-related rehospitalization and last medical contact in our clinic over a follow-up period of at least 5 years. Secondly, family physicians of those patients with incomplete follow-up after step 1 (i.e. either patients without any re-admission or patients re-admitted for the last time before expiration of total 5 years follow-up) were contacted to complete survival status. Thirdly, survival status was completed by individual telephone visits with the remaining patients, who did not complete total 5 years follow-up after follow-up steps 1 and 2.

### Statistical methods

For normally distributed data, the Student *t* test was applied. Otherwise, the Mann–Whitney *U* test was used as nonparametric test. Deviations from a Gaussian distribution were tested by the Kolmogorov-Smirnov test. hFABP and NT-proBNP data were log_10_ transformed, thereby promoting normality, and the unpaired *t*-test was applied. Spearman’s rank correlation for nonparametric data was used to test the association of hFABP blood levels with clinical parameters. Qualitative parameters were analyzed by use of a 2 × 2 contingency table and Chi^2^ test or Fisher’s exact test as appropriate. Quantitative data are presented as mean ± standard error of mean (SEM) or as median and interquartile range (IQR), depending on the distribution of the data. For qualitative parameters absolute and relative frequencies are presented. All analyses were exploratory and utilized a p value of 0.05 (2 tailed) for significance.

### Diagnostic value of combined hFABP plus NT-proBNP versus NT-proBNP alone

1) C-statistics: Receiver-operating characteristic curve (ROC) analyses with areas under the curves (AUC) were calculated for diagnosis of AHF. Two logistic regression models with AHF as dependent variable and the combined biomarkers hFABP plus NT-proBNP as well as NT-proBNP alone (reference biomarker) as independent variables were analyzed. The two areas under the ROC curves were compared using the method by Hanley [38]. The optimal cutoff for the combined biomarkers accorded to the probabilities being associated with hFABP and NT-proBNP to suffer from AHF as assessed by logistic regression models and was chosen where the Youden index (sensitivity + specificity − 1) yielded maximum values. Optimal cutoffs of single NT-proBNP measurements (i.e. reference) accorded to <300 pg/ml to “rule out AHF”, as well as to “rule-in AHF” according to age-dependent cutoff levels of 450 ng/l (age <50 years), 900 ng/l (50–75 years), and 1800 ng/l (>75 years) [24]. Contingency tables were used to assess the individual diagnostic goodness criteria (i.e. accuracy, specificity, sensitivity, negative/positive predictive values (NPV/PPV)). Statistical accuracy was defined as the sum of true positives plus true negatives devided by the number of total measurements (*n* = 401). Accuracy, specificity, sensitivity were compared by McNemar tests.

### Prognostic value of hFABP versus troponin I

Kaplan Meyer curves according to hFABP quartiles were created and the corresponding hazard ratios (HRs) were calculated for each hFABP quartile in all patients for the two mentionend prognostic outcomes at 1 and 5 years (i.e. all cause mortality and AHF related rehospitalization).

Prognostic models were performed using Cox regression analyses including the following 12 variables to a reference model [39]: age, sex, left ventricular function, serum creatinine (mg/dl), NYHA functional class, presence of diabetes mellitus, the degree of coronary artery disease, haemoglobin (g/dl), serum sodium (mmol/l), beta-blocker treatment, angiotensin-converting enzyme inhibitor or angiotensin II receptor blocker treatment (ACEI/ARB) and logNT-proBNP levels. Variables of the reference model were chosen either because of univariate significant associations or because of commonly known prognostic effects [23, 39].

The biomarkers of interest, hFABP and troponin I, were subsequently added to this reference model as continuous variables.

To test whether the inclusion of hFABP and troponin I valuably improves prognosis of the reference model for all-cause mortality and AHF related rehospitalization, different established measurements of model performance were applied [23, 39–41].

Statistical measures of discrimination comprised C-statistics. C-statistics of models with additional hFABP or troponin I were compared using the Mann–Whitney *U* test. Statistical measures of model calibration comprise Hosmer-Lemeshow test, Bayesian information criterion (BIC), Akaike information criterion (AIK) and the Brier score. The global goodness of fit of the models was evaluated by likelihood ratio tests. Statistical measures of reclassification comprise integrated discrimination improvement (IDI) and net reclassification improvement (NRI) following Pencina [42].

In Cox regression models an increase of hazard ratio (HR) correspond to the logarithmic function of NT-proBNP (ng/l), hFABP per every 10 ng/ml and TnI per every μg/l change.

The calculations were performed with InStat and StatMate (GraphPad Software), SPSS software (SPSS Software GmbH), and SAS (SAS Institute Inc. Cary, NC, USA).

## Results

### Associations of hFABP levels with clinical parameters

**Fig. 1:**
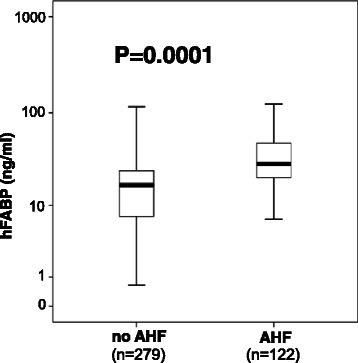
hFABP levels were significantly higher in patients suffering from acute heart failure (*AHF*) (*n* = 122) compared to those without (*n* = 279) (*p* = 0.0001). Data are presented as medians with 25th and 75th percentiles (boxes) and 5th and 95th percentiles (whiskers)

**Fig. 2:**
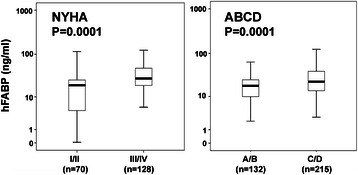
hFABP levels were significantly higher in patients of functional NYHA class III/IV (*n* = 128) compared to those of NYHA class I/II (*n* = 70) (*p* = 0.01) (*left*) and higher in patients of structural AHA/ACC stage C/D (*n* = 215) compared to those of stage A/B (*n* = 132) (*p* = 0.0001) (*right*). Data are presented as medians with 25th and 75th percentiles (boxes) and 5th and 95th percentiles (whiskers)

Baseline characteristics of 401 study patients are summarized in Table 1. hFABP levels were able to differentiate patients with AHF (median = 28.8 ng/ml, IQR 20.3–48.1 ng/ml, *n* = 122) from those without (median = 16.9 ng/ml, IQR 7.2–24.4 ng/ml, *n* = 279) (*p* = 0.0001) (Fig. 1). hFABP levels were higher in NYHA class III/IV patients compared to NYHA class I/II patients (NYHA III/IV: median = 27.5 ng/ml, IQR 18.8–48.0 ng/ml, *n* = 128; NYHA I/II: median = 19.1 ng/ml, IQR 3.6–25.5 ng/ml, *n* = 70) (*p* = 0.0001) (Fig. 2, left) and higher in ACC/AHA class C/D patients compared to class A/B patients (class D/E: median = 22.6 ng/ml, IQR 13.8–39.2 ng/ml, *n* = 215; class A/B: median = 18.1 ng/ml, IQ range 9.8–25.1 ng/ml, *n* = 132) (*p* = 0.0001) (Fig. 2, right). Patients with LV dysfunction being assessed by echocardiography (i.e. LVEF <55 %) had significantly higher levels of hFABP than patients with regular heart function (median = 27.0 ng/ml, IQR 17.3–50.9, *n* = 91 versus 19.2 ng/ml, 11.1–28.2, *n* = 135) (*p* = 0.0001). hFABP levels correlated significantly (*p* < 0.05) with several indices of cardiac function being assessed by echocardiography, such as fractional shortening (*r* = −0.20), E/A (*r* = 0.23), left atrial diameter (*r* = 0.29), LV enddiastolic diameter (*r* = 0.34) and LV endsystolic diameter (*r* = 0.29). hFABP serum levels correlated significantly (*p* < 0.05) with creatinine (*r* = 0.22), hemoglobin (*r* = −0.13), NT-proBNP (*r* = 0.32) and TnI levels (*r* = 0.23).

**Table 1:** Baseline characteristics of 401 patients initially presenting with acute dyspnea or peripheral edema

Variables	All patients	No AHF	AHF	*p* value*
	(*n* = 401)	(*n* = 279)	(*n* = 122)	
Demographics				
Age, mean (range)	67 (18–96)	65 (18–96)	73 (36–96)	0.0001
Male, *n* (%)	205 (51)	131 (47)	74 (61)	0.7
Cardiovascular risk factors, *n* (%)				
Arterial hypertension	268 (67)	170 (61)	98 (80)	0.0001
Hypercholesterinemia	122 (30)	207 (74)	72 (59)	0.003
Cardiac family history	132 (33)	97 (35)	35 (27)	0.3
Smoking	206 (51)	148 (53)	58 (48)	0.3
Diabetes mellitus	120 (30)	67 (24)	53 (43)	0.0001
Main diagnoses, *n* (%)				
Chronic CHF	143 (36)	63 (23)	80 (66)	0.0001
Atrial fibrillation	90 (22)	40 (14)	50 (41)	0.0001
Coronary artery disease	130 (32)	73 (26)	57 (47)	0.0001
Prior myocardial Infarction	89 (22)	47 (17)	42 (34)	0.0002
Valvular heart disease	118 (29)	48 (17)	70 (57)	0.0001
Acute exacerbated COPD	31 (8)	27 (10)	4 (3)	0.03
Acute exacerbated asthma	7 (2)	7 (3)	0 (0)	0.1
Pneumonia	20 (5)	19 (7)	1 (1)	0.01
Pulmonary embolism	12 (3)	12 (4)	0 (0)	0.02
Chronic kidney disease	71 (17)	30 (11)	41 (34)	0.0001
Cancer	19 (5)	19 (7)	0 (0)	0.001
Stroke	10 (2)	8 (3)	2 (2)	0.7
LV EF (%), median (interquartile range)	41 (30–52)	50 (40–58)	39 (27–50)	0.04
Symptoms and signs, *n* (%)				
Peripheral edema	46 (11)	42 (15)	4 (3)	0.02
Dyspnea	235 (59)	183 (66)	52 (42)	0.0001
Both peripheral edema and dyspnea	120 (30)	54 (19)	66 (54)	0.0001

*p values for the comparison between AHF and no AHF group

### Diagnostic value of hFABP in combination with NT-proBNP

The AUC for the combined biomarkers hFABP plus NT-proBNP was equal to the AUC of NT-proBNP alone (hFABP plus NT-proBNP, AUC: 0.85; 95 % CI 0.79–0.87; NT-proBNP, AUC: 0.85; 95 % CI 0.81–0.89; AUC difference 0.0; *p* > 0.05), demonstrating that both AUCs valuable disriminate AHF patients from no AHF patients. The AUC of hFABP alone for diagnosis of AHF was 0.72 (95 % CI 0.67–0.78, *p* = 0.0001).

Combining hFABP plus NT-proBNP compared to single NT-proBNP at the “rule out AHF” cutoff of <300 ng/l revealed a significant increase in specificity, statistical accuracy and PPV (*p* = 0.0001): specificity (combined: 71 %, 95 % CI 66–77 %; NT-proBNP alone: 48 %, 95 % CI 42–54 %), PPV (combined: 58 %, 95 % CI 51–65 %; NT-proBNP alone: 45 %, 95 % CI 39–51 %), statistical accuracy (combined: 77 %, 95 % CI 73–81 %; NT-proBNP alone: 63 %, 95 % CI 58–68 %). Sensitivity (combined: 89 %, 95 % CI 84–95 %; NT-proBNP: 96 %, 95 % CI 92–99 %) and NPV (combined: 94 %, 95 % CI 91–97 %; NT-proBNP: 96 %, 95 % CI 93–99 %) were slightly decreased (at least 7 %).

Evaluation of hFABP plus NT-proBNP compared to NT-proBNP alone according to age-dependent cutoff levels to “rule-in AHF” (450 ng/l (age <50 years), 900 ng/l (50–75 years), and 1800 ng/l (>75 years)) did not reveal any improvements neither in AUC’s, sensitivities, specificities, statistical accuracy nor predictive values (data not shown).

### Prognostic value of hFABP compared to troponin I

Non-survivors (*n* = 57) had a median hFABP value of 28.6 ng/ml (IQR 16.2–49.1 ng/ml) vs. 19.0 ng/ml (IQR 10.7–28.3 ng/ml) in survivors (*p* = 0.0009) at 1 year, and 26.2 pg/ml (IQR 16.2–44.9) in non-survivors (*n* = 129) vs. 17.8 ng/ml (IQR 10.3–24.9 ng/ml) in survivors at 5 years respectively (*p* = 0.0001). Patients being rehospitalized because of AHF had significantly higher hFABP levels than patients not being rehospitalized (1 year: AHF related rehospitalization, *n* = 34, median = 39.7 ng/ml (IQR 23.8–77.2 ng/ml), no AHF related rehospitalization, *n* = 367, median = 27.5 ng/ml (IQR 18.2–47.2 ng/ml), *p* = 0.0001; 5 years: AHF related rehospitalization, *n* = 73, median = 27.5 ng/ml (IQR 18.2–47.2 ng/ml), no AHF related rehospitalization, *n* = 328, median = 18.9 ng/ml (IQR 10.2–28.3 ng/ml), *p* = 0.0001).

### Kaplan Meier survival analyses

**Fig. 3:**
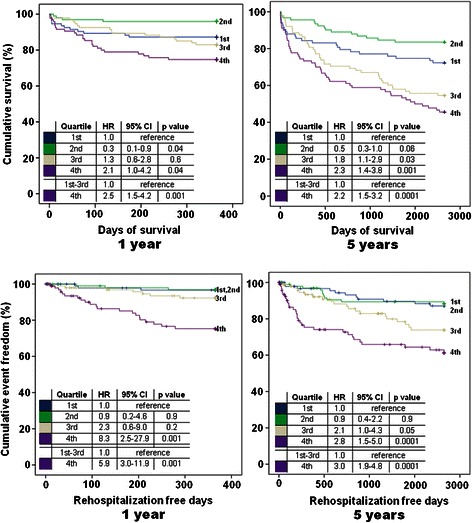
Kaplan-Meier curves evaluated by quartiles of hFABP after 1 (*left panel*) and 5 (*right panel*) years of follow-up in the total study cohort (*n* = 401). Increasing hFABP levels were significantly associated with long term all-cause mortality (**a**, *top*) and AHF related rehospitalization (**b**, *bottom*). Hazard Ratios (*HR*) were calculated for each risk group according to hFABP quartiles

Kaplan Meier survival curves illustrate increasing risk of all-cause death at 1 and 5 years according to quartiles of hFABP. Patients with highest hFABP levels of the 4th quartile were up to 2.5 times more likely to die within follow-up periods (range of HRs: 2.1–2.5; *p* = 0.04) (Fig. 3, top). Additionally, patients with highest hFABP levels of the 4th quartile were up to 8.3 times more likely to be rehospitalized because of AHF within follow up periods (range of HRs 2.8–8.3, *p* = 0.001) (Fig. 3, bottom).

### Performance metrics of prognostic models

#### Discrimination

**Fig. 4:**
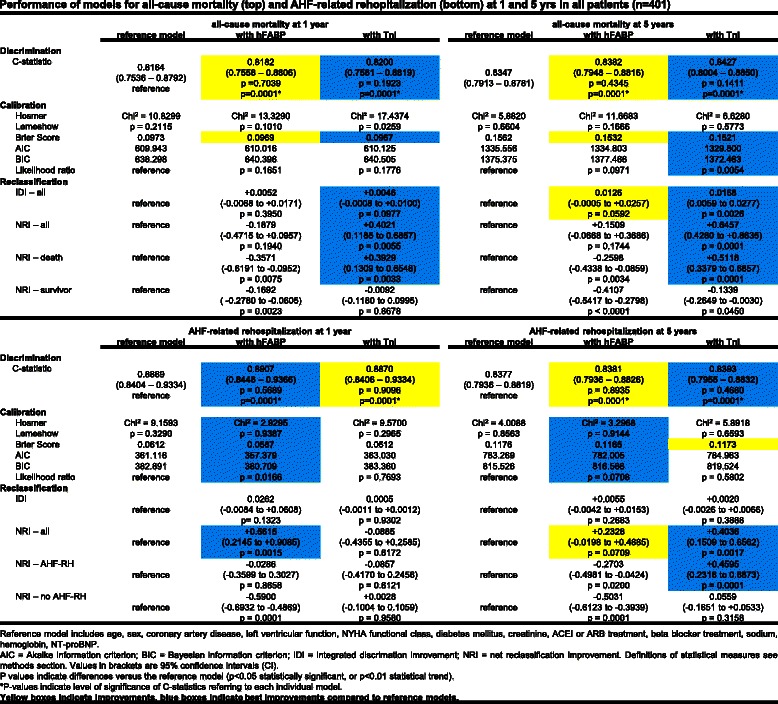
Performance of models for all-cause mortality (*top*) and AHF-related rehopitalization (*bottom*) at 1 and 5 years in all patients (*n* = 401)

**Fig. 5:**
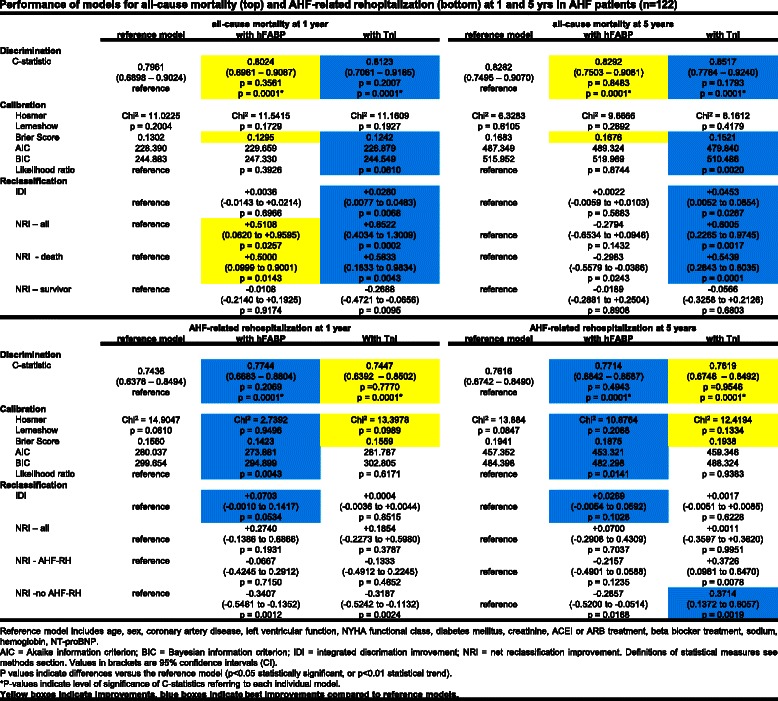
Performance of models for all-cause mortality (*top*) and AHF-related rehopitalization (*bottom*) at 1 and 5 years in AHF patients (*n* = 122)

Table 2 shows univariable associations of all variables with the prognostic outcomes. Either hFABP or TnI were adjusted in multivariable prognostic models relative to conventional assessment including 11 risk factors plus NT-proBNP. All-cause mortality and AHF related rehospitalization were significantly discriminated by including hFABP or TnI either in all (*n* = 401) or in AHF patients (*n* = 122), as indicated by individual significant AUCs above 0.7 (*p* = 0.0001, referring to each individual model) (Figs. 4 & 5).

**Table 2:** Significance (*p* values) of univariate associations of prognostic variables for all-cause mortality and AHF related rehospitalization in all patients (*n* = 401)

	All-cause mortality		AHF related rehospitalization	
	1 year	5 years	1 year	5 years
Sex	0.7174	0.9621	0.4660	0.9865
Age	0.0001*	0.0001*	0.0007*	0.0001*
Diabetes	0.9756	0.3776	0.0030*	0.0001*
Left ventricular function	0.0088*	0.0002*	0.0001*	0.0001*
NYHA class	0.2061	0.0029*	0.0001*	0.0001*
Coronary artery disease	0.5443	0.4715	0.0203*	0.0001*
Beta blocker	0.9888	0.9876	0.0965	0.0008*
ACE inhibitor	0.6988	0.2305	0.0002*	0.0001*
Hemoglobin	0.0001*	0.0001*	0.0182*	0.1096
Creatinine	0.0554	0.0032*	0.0001*	0.0001*
Sodium	0.0661*	0.0099*	0.3777	0.7839
NTproBNP	0.0001*	0.0001*	0.0001*	0.0001*
hFABP	0.0008*	0.0001*	0.0001*	0.0001*
Troponin I	0.0009*	0.0001*	0.0001*	0.0002*

*Level of significance, *p* < 0.05

When compared to the reference model, prognostic models including TnI revealed only numerically greatest, but not significantly different AUCs for all-cause mortality when applied in all (*n* = 401) as well as in AHF patients (*n* = 122) at 1 and 5 years (highlighted in blue, Figs. 4 & 5, top).

Accordingly, prognostic models including hFABP revealed only numerically greatest, but not significantly different AUCs for AHF-related rehospitalization when applied in all and AHF patients at 1 and 5 years (exception in all patients at 5 years) (highlighted in blue, Figs. 4 & 5, bottom).

### Calibration and reclassification

All-cause mortality was best calibrated in prognostic models including TnI (except all-cause mortality at 1 years in all patients). These models revealed favourable measures of calibration (such as Hosmer Lemshow, Brier Score, AIC, BIC and likelihood ratios) as well as beneficial NRIs (Figs. 4 & 5, top, highlighted in blue). AHF related rehospitalization was best calibrated in prognostic models including hFABP measurements. These models revealed favourable measures of calibration, such as Hosmer Lemshow, Brier Score, AIC, BIC and likelihood ratios as well as beneficial NRIs (Figs. 4 and 5, bottom, highlighted in blue). In contrast, IDIs mostly revealed only low values and indicated none or insubstantial integrative improvements.

### Cox regression analyses

Multivariate prognostic Cox proportional hazard models, incorporating the same 12 risk factors as described above, revealed up to 2 % increase of all-cause mortality risk at 5 years alongside with increasing hFABP levels of 10 ng/ml being initially measured only once in the emergency department in all patients (HR 1.020; 95 % CI 1.020–0.998; statistical trend *p* < 0.1) (Table 3). In contrast, increasing TnI levels of 1 μg/l were not associated with an increased risk of all-cause mortality (*p* > 0.1) (Tables 3 and 4).

**Table 3:** Multivariable Cox Regression analyses for all-cause mortality and AHF related rehospitalization at 5 years in all patients (*n* = 401)

	All-cause mortality						AHF-related reospitalization					
	hFABP			Troponin I			hFABP			Troponin I		
	HR	95 % CI	*p* Value	HR	95 % CI	*p* Value	HR	95 % CI	*p* Value	HR	95 % CI	*p* Value
Age	1.033	1.016–1.050	**0.001**	1.036	1.019–1.054	**0.001**	1.012	0.989–1.034	0.301	1.012	0.989–1.034	0.296
Sex	1.261	0.844–1.884	0.259	1.193	0.788–1.804	0.405	1.012	0.593–1.726	0.966	0.989	0.574–1.705	0.968
NYHA functional class	1.107	0.826–1.485	0.496	1.091	0.816–1.458	0.556	1.799	1.257–2.576	**0.001**	1.765	1.236–2.519	**0.002**
Left ventricular function	1.227	0.964–1.562	0.096	1.267	0.993–1.616	0.057	1.013	0.756–1.359	0.930	0.995	0.745–1.329	0.973
Coronary artery disease	0.843	0.555–1.280	0.422	0.907	0.595–1.380	0.648	1.438	0.824–2.509	0.201	1.469	0.839–2.569	0.178
Diabetes mellitus	1.194	0.798–1.786	0.389	1.113	0.747–1.660	0.598	1.757	1.056–2.924	**0.030**	1.623	0.979–2.693	0.061
Creatinine (mg/dl)	0.783	0.470–1.305	0.348	0.849	0.515–1.398	0.519	0.797	0.414–1.533	0.496	0.859	0.451–1.636	0.643
Sodium (mmol/l)	0.974	0.939–1.011	0.160	0.976	0.941–1.012	0.183	1.039	0.978–1.104	0.212	1.039	0.978–1.103	0.218
Hemoglobin (g/dl)	0.860	0.776–0.954	**0.005**	0.868	0.782–0.962	**0.007**	1.000	0.874–1.145	0.997	0.996	0.870–1.141	0.957
ACEI / ARB treatment	0.707	0.478–1.045	0.082	0.714	0.484–1.052	0.089	1.647	0.969–2.799	0.066	1.651	0.967–2.820	0.066
Beta-blocker treatment	0.844	0.559–1.275	0.421	0.853	0.567–1.282	0.443	1.066	0.629–1.805	0.813	1.044	0.616–1.769	0.873
LogNT-proBNP (ng/l)	1.445	1.254–1.665	**0.001**	1.525	1.325–1.755	**0.001**	1.209	0.993–1.472	0.058	1.251	1.027–1.523	**0.026**
hFABP (10 ng/ml)	1.020	1.020–0.998	0.081	-	-	-	1.034	1.001–1.069	**0.047**	-	-	-
Troponin I (μg/l)	-	-	-	0.857	0.696–1.057	0.148	-	-	-	0.982	0.914–1.055	0.620

The logarithmic function of NT-proBNP and hFABP per every 10 ng/ml change were used in Cox Models. Significant p values (*p*<0.05) are written in bold type. Dashes indicate non applicable

*HR* hazard ratio, *CI* confidence interval

**Table 4:** Multivariable Cox Regression analyses for all-cause mortality and AHF related rehospitalization at 5 years in AHF patients (*n* = 122)

	All-cause mortality						AHF-related reospitalization					
	hFABP			Troponin I			hFABP			Troponin I		
	HR	95 % CI	*p* Value	HR	95 % CI	*p* Value	HR	95 % CI	*p* Value	HR	95 % CI	*p* Value
Age	1.056	1.025–1.088	**0.001**	1.060	1.029–1.092	**0.001**	1.027	0.997–1.058	0.079	1.032	1.002–1.062	**0.039**
Sex	1.096	0.573–2.098	0.783	0.657	0.317–1.362	0.259	1.354	0.651–2.815	0.418	1.548	0.742–3.230	0.244
NYHA functional class	0.942	0.619–1.433	0.780	0.501	0.075–3.333	0.475	1.610	1.001–2.592	**0.049**	0.798	0.059–10.61	0.864
Left ventricular function	1.263	0.926–1.724	0.141	1.450	1.040–2.021	**0.028**	1.227	0.875–1.720	0.236	1.167	0.836–1.628	0.364
Coronary artery disease	0.812	0.423–1.561	0.532	0.893	0.461–1.727	0.736	1.140	0.559–2.323	0.718	1.133	0.558–2.302	0.731
Diabetes mellitus	1.163	0.622–2.178	0.635	1.172	0.625–2.198	0.620	1.324	0.682–2.570	0.408	1.202	0.623–2.317	0.584
Creatinine (mg/dl)	0.952	0.463–1.954	0.892	1.234	0.597–2.547	0.571	0.400	0.159–1.003	0.051	0.448	0.187–1.073	0.072
Sodium (mmol/l)	0.978	0.920–1.039	0.463	0.967	0.910–1.026	0.264	1.113	1.023–1.211	**0.013**	1.123	1.030–1.225	**0.008**
Hemoglobin (g/dl)	0.836	0.701–0.998	0.047	0.827	0.694–0.986	0.034	0.949	0.792–1.139	0.576	0.934	0.780–1.117	0.453
ACEI/ARB treatment	0.919	0.498–1.701	0.790	1.005	0.544–1.859	0.987	1.425	0.722–2.812	0.307	1.501	0.758–2.974	0.244
Beta-blocker treatment	0.895	0.488–1.644	0.721	0.826	0.451–1.511	0.535	1.387	0.704–2.732	0.345	1.550	0.795–3.023	0.198
LogNT-proBNP (ng/l)	1.207	0.911–1.601	0.190	1.324	0.997–1.757	0.052	0.911	0.696–1.192	0.496	1.015	0.780–1.320	0.912
hFABP (10 ng/ml)	0.996	0.948–1.047	0.873	-	-	-	1.073	1.018–1.132	**0.008**	-	-	-
Troponin I (μg/l)	-	-	-	0.789	0.567–1.071	0.125	-	-	-	0.982	0.914–1.055	0.620

The logarithmic function of NT-proBNP and hFABP per every 10 ng/ml change were used in Cox Models. Significant p values (p<0.05) are written in bold type. Dashes indicate non applicable

*HR* hazard ratio, *CI* confidence interval

Accordingly, Cox models revealed up to 3–7 % increase of AHF related rehospitalization risk at 5 years alongside with increasing hFABP levels of 10 ng/ml (all patients: HR 1.034; 95 % CI 1.001–0.068; *p* = 0.047; AHF patients: HR 1.073; 95 % CI 1.018–0.132; *p* = 0.008). In contrast, increasing TnI levels of 1 μg/l were not associated with an increased risk of AHF related rehospitalization (Tables 3 and 4).

## Discussion

This post-hoc analysis of the MANPRO study evaluated the diagnostic and long-term prognostic values of hFABP levels in comparison to NT-proBNP and TnI in patients presenting to the emergency department with acute dyspnea and peripheral edema being suspected of acute heart failure (AHF). To the best of our knowledge, the present analysis is the first investigating the diagnostic and prognostic value of hFABP measurements in patients being suspected of AHF.

hFABP levels were able to differentiate AHF from other causes of dyspnea and/or edema, were associated with higher disease stages of CHF and correlated significantly with several indices of cardiac function being assessed by echocardiography. Combining hFABP with NT-proBNP increased specificity, statistical accuracy and PPV when compared to the age-independent cutoff of single NT-proBNP <300 pg/ml to “rule-out AHF”. Secondly, highest hFABP levels measured initially in the emergency department were associated with all-cause mortality and AHF related rehospitalization at 1 and 5 years as assessed by Kaplan-Meyer analyses. Within multivariate adjustment in prognostic models containing 11 conventional prognostic risk factors plus NT-proBNP, all-cause mortality was better characterized in terms of statistical calibration and reclassification by additionally including TnI. In contrast, AHF-related rehospitalization was better characterized in multivariate models including hFABP.

In the context of heart failure syndromes, hFABP has mostly been evaluated in studies including adolescent patients suffering from CHF, thereby commonly focusing on the prognostic value of hFABP to predict both mortality and adverse cardiac events [43–45]. In contrast, increasing hFABP levels were shown to be associated with the severity of heart failure in children with chronic endocardial fibroelastosis or dilated cardiomyopathy [25]. Additionally it has been demonstrated that hFABP was even more sensitive than brain natriuretic peptide (BNP) to detect the development of secondary AHF in children primarily suffering from pneumonia [28].

The present study focused on the improvement of the weaknesses of the age-independent cutoff value of NT-proBNP <300 ng/l to “rule out AHF”, such as the low specificity, accuracy and PPV [29]. As recently published for the presented study cohort [29], the NT-proBNP cutoff to “rule out AHF” was set at <300 ng/l with a corresponding sensitivity of 96 %, specificity of 48 %, PPV of 45 % and a NPV of 96 %.

As demonstrated in this study, increasing the specificity by additional hFABP measurements might detect even more patients as “no AHF”, who truly do not suffer from AHF although presenting with dyspnea and/or peripheral in the emergency department. In contrast, additional hFABP measurements were not shown to reveal any improvements when applied for age-dependant NT-proBNP cutoffs to “rule in AHF” [24].

The usefulness of hFABP as a biomarker for the detection of AHF is mainly driven by the molecule’s biochemistry and pathophysiological behavior during myocardial injury. With a molecular size of 15 kDa hFABP is a small cytosolic protein, being highly present in the myocardium compared to skeletal muscle [4]. Besides this relative tissue specificity, hFABP is rapidly released into the serum within 2 h after myocardial injury, whereas it is usually not present in serum under healthy conditions. The development of acute heart failure is facilitated by myocardial ischemia and necrosis, cardiomyocyte damage from inflammatory cytokines, oxidative stress or apoptosis, thereby increasing the permeability of the cardiomyocyte membrane with consecutive release of cytosolic proteins [31, 46]. These conditions might also be the main stimulus for hFABP serum release in AHF [5, 10]. Therefore measurements of hFABP might be worth to be tested in a setting of patients being suspected of acute heart failure [31].

It is assumed that hospitalization because of recurrent AHF occurs in up to 30 % of heart failure patients within 90 % days post-discharge [47] and therefore has a major impact on the health care system. Conflicting data are available about the effectiveness of discharge planning, disease management programs and tele-monitoring in heart failure patients, whereas it remains unclear, whether AHF readmissions are preventable and do necessarily indicate a suboptimal standard of care [47]. Yet the economic benefit implementing hFABP into clinical risk stratification has not been evaluated at all. Additional costs due to the implementation of a new prognostic biomarkers can only be justified by an improvement of clinical outcomes themselves [23, 48, 49].

Based on our findings, the prognostic value of hFABP next to troponin I and NT-proBNP testing might identify those patients with an increased risk of upcoming AHF related rehospitalization or death. Within the present study, it was demonstrated that prognosis of all-cause mortality was better reclassified according to improved net reclassification improvements (NRI) due to additional measurements of TnI. Interestingly, patients without a future AHF related rehospitalization were more correctly reclassified due to additional measurements of hFABP. Each unit increase of hFABP by 10 ng/ml being measured only once in the emergency department was associated with a 2 % increase of all-cause mortality and 3–7 % increase of AHF related rehospitalization risk at 5 years. All prognostic models were calculated to current state-of-the-art statistics in order to receive valuable generalizing results [23, 39–41].

Therefore, our findings might help to further improve risk stratification of patients being suspected of AHF in order to reduce heart failure related mortality, readmissions and possibly health care costs after an initial emergency presentation and in-hospital treatment because of AHF [47, 48, 50, 51].

### Study limitations

The present study was conducted as a post-hoc non-randomized single-center study. The primary inclusion criteria have been patients presenting with acute dyspnea and/or peripheral edema to the emergency department. AHF diagnosis was only made by one independent cardiologist as stated, while a triple panelist approach might have further increased the validity of AHF diagnosis [29]. Initial medical heart failure treatment (e.g. by nitrates and diuretics) might have influenced hemodynamic changes, the relief of clinical symptoms and heart function over time. Evaluable echocardiographic examinations were available in 210 of 401 patients with a median of 3 days after the inital emergency department presentation [30]. Assessment of hFABP performance in patients with HFpEF versus HFrEF has been beyond the scope of the present analysis. Patients with a history of cerebro-vascular diseases (CVA or TIA) and atrial fibrillation were not excluded and might have influenced the range of hFABP levels. Cardiovascular mortality was not evaluated as a prognostic outcome in the present trial. Only index-hospital AHF-related rehospitalization was assessed. Competing AHF related rehospitalization at other hospitals was not evaluated. Prognostic event rates might have been under-estimated and might not reflect the complete clinical reality. Evaluating our results with ultra-sensitive troponin methods may allow better diagnostic and prognostic results compared to the contemporary TnI assay being used in this study. Notably, hFABP was measured by a manual, not standardized immunoassay, which might be time consuming and not cost effective in current clinical practice, as there is no automated bedside test currently available on the market. The diagnostic and prognostic values of hFABP, as well as its standardized and cost-effective measurement need to be confirmed by ongoing medical research within larger prospective clinical studies with AHF patients specifically taking into account the above mentioned limitations.

## Conclusions

Taken together, this post-hoc analysis demonstrates that (1) additional hFABP measurements improved diagnostic specificity of sole NT-proBNP testing at the cutoff <300 ng/l to “rule out” AHF. (2) In prognostic models containing 11 conventional risk factors plus NT-proBNP, hFABP mostly improved prognostic models for AHF-related rehospitalization at 1 and 5 years, whereas troponin I mostly improved prognostic models for all-cause mortality.
